# The RAGE/multiligand axis: a new actor in tumor biology

**DOI:** 10.1042/BSR20220395

**Published:** 2022-07-01

**Authors:** Armando Rojas, Ivan Schneider, Cristian Lindner, Ileana Gonzalez, Miguel A. Morales

**Affiliations:** 1 Biomedical Research Labs., Universidad Catolica del Maule, Facultad de Medicina, 3605 San Miguel Ave., Talca, Chile; 2 Department of Molecular and Clinical Pharmacology Program, Institute of Biomedical Sciences, Universidad de Chile, Santiago 8320000, Chile, Santiago, Chile

**Keywords:** advanced glycation, alarmins, receptor advanced glycation end-products, tumor biology, tumor microenvironment

## Abstract

The receptor for advanced glycation end-products (RAGE) is a multiligand binding and single-pass transmembrane protein which actively participates in several chronic inflammation-related diseases. RAGE, in addition to AGEs, has a wide repertoire of ligands, including several damage-associated molecular pattern molecules or alarmins such as HMGB1 and members of the S100 family proteins.

Over the last years, a large and compelling body of evidence has revealed the active participation of the RAGE axis in tumor biology based on its active involvement in several crucial mechanisms involved in tumor growth, immune evasion, dissemination, as well as by sculpturing of the tumor microenvironment as a tumor-supportive niche. In the present review, we will detail the consequences of the RAGE axis activation to fuel essential mechanisms to guarantee tumor growth and spreading.

## Introduction

Tumor biology is characterized by a complex spectrum of alterations, ranging from aberrant intracellular events to those involving communication between cells and with neighboring tissues. In this setting, the tumor microenvironment (TME) is a dynamic niche where complex and reciprocal interactions are established, not only among cancer cells but also with a myriad of infiltrating immune and stromal cells, as well as the surrounding extracellular matrix (ECM) [1].

Under healthy conditions, pro- and anti-inflammatory signals are maintained in a state of balance, referred to as inflammatory homeostasis [2]. Dysregulation of this balance leads to the onset and development of numerous human diseases, including cancer, where chronic inflammation is a widely recognized contributor to shaping a supportive microenvironment for tumor growth and development [3–5].

The protein, receptor for advanced glycation end-products (RAGE), was initially reported as the receptor for advanced glycation end-products (AGEs) [6], which are a broad and heterogeneous group of compounds derived from structural modifications of proteins, lipids, and nucleic acids, which become non-enzymatically glycated by reducing sugars [7]. Although these compounds were originally described in the well-known Maillard reaction, AGEs are also endogenously synthesized by a non-enzymatic reaction involving a glycation/condensation process between reducing sugars, such as glucose and fructose, and the free amino group of different biomolecules, such as proteins, lipids, and nucleic acids, to initially form Schiff bases, which are subsequently converted to intermediate glycation products, known as Amadori products [8].

Degradation of Schiff bases and Amadori products generates highly reactive short-chain carbonyl compounds, known as α-dicarbonyls, or α-oxaldehydes, such as oxoaldehydes glyoxal, methylglyoxal (MGO), and 3-deoxyglucosone, which can also be formed during glycolysis, as well as by glucose autoxidation in the presence of catalytic metals [9].

**Figure 1:**
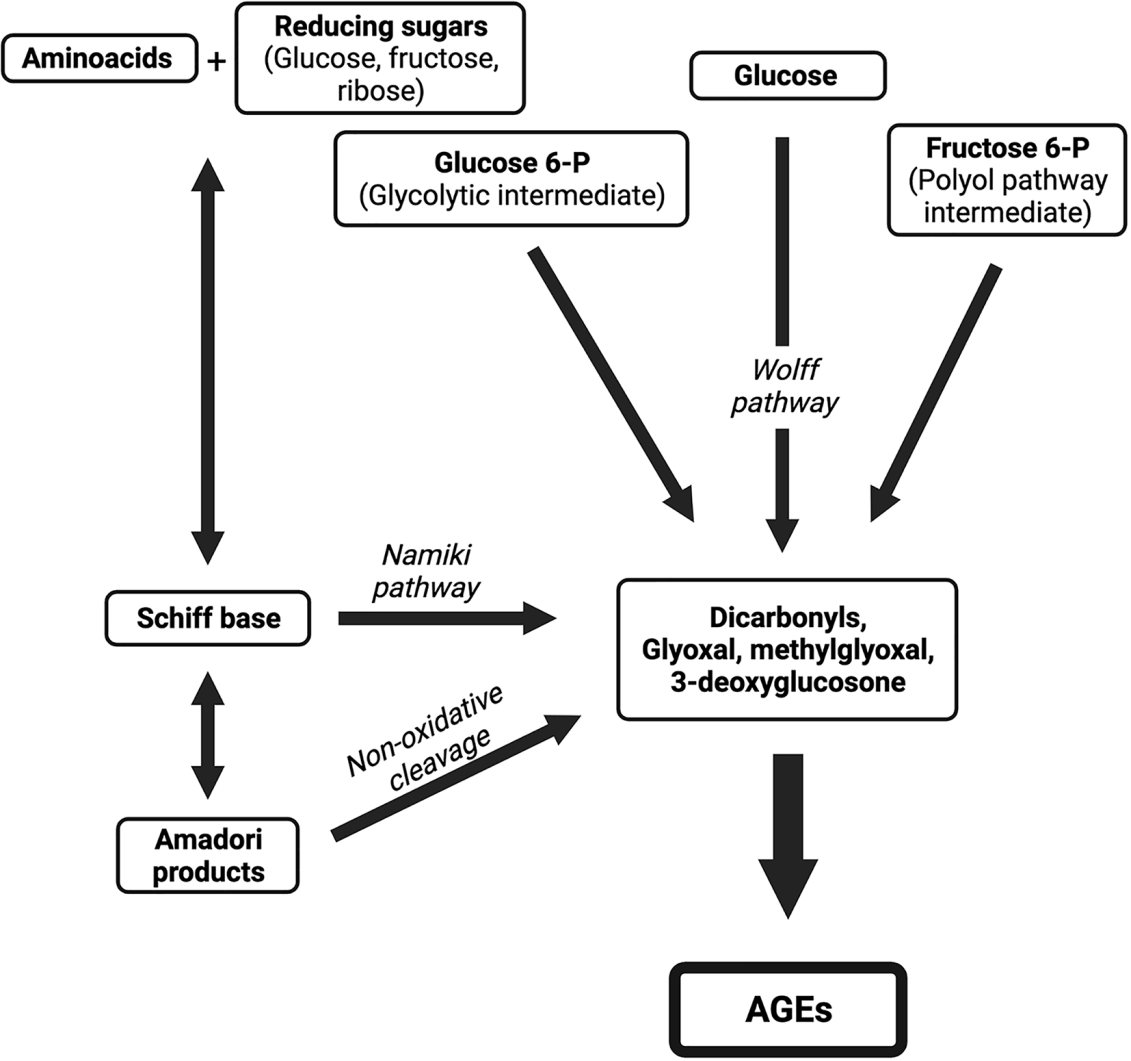
Different pathways are involved in advanced glycation end-product formation AGEs are endogenously synthesized by a non-enzymatic reaction involving a glycation/condensation process between reducing sugars and the free amino group of different biomolecules to form Schiff bases, which are subsequently converted to Amadori products. Degradation of both Schiff bases (Nakimi pathway) and Amadori products (non-oxidative cleavage) renders the main intermediate compounds in the formation of AGEs. These highly reactive intermediates are also formed by other metabolic intermediates

These α-dicarbonyl compounds are highly reactive, and participate in the formation of intra- or inter-protein cross-links. The production of α-dicarbonyls proceeds by different pathways, including the Namiki pathway, when a Schiff base degrades and forms glyoxal, and the Wolff pathway, which involves the autoxidation of monosaccharides. In addition, other metabolic intermediates have been implicated in α-dicarbonyl production, including glycolytic intermediates (glucose-6-phosphate, glyceraldehyde-3-phosphate, and dihydroxyacetone phosphate) and fructose-6-phosphate (polyol pathway intermediate) [10,11] (Figure 1).

Data raised from both *in vitro* and *in vivo* studies supported that MGO-driven biological activities are positively related to cancer onset and progression. MGO is considered a genotoxic agent based on its capacity to produce oxidative damage to DNA and DNA adducts as well [12,13]. MGO-modified proteins, such as heat shock protein (Hsp) are responsible to protect cancer cells from apoptosis, as reported in non-small cell lung cancer and gastrointestinal neoplasia [14,15]. Furthermore, accumulation of MGO adducts are associated with tumor aggressiveness in colorectal cancer [16].

Although AGEs were initially recognized as formed in excess in diabetes, due to hyperglycemia, exogenous AGEs, mostly derived from dietary intake, are important contributors to the physiological AGEs pool [11,17]. In this setting, emerging data support a potential role for high dietary AGEs intake in human carcinogenesis, particularly of gallbladder cancer, because of their pro-inflammatory and pro-oxidative properties [18].

The formation of AGEs also occurs in cancer cells through the ‘Warburg effect’, which leads to increased production of MGO, a reactive dicarbonyl known to be the major precursor of AGEs. This increased formation and accumulation of MGO in cancer cells promotes tumor development and progression [19–21].

Soon afterward its discovery, the binding capacity of RAGE was found to be extended to other ligands, beyond AGEs, such as the alarmin, high mobility group box 1 (HMGB1), and members of the S-100 calgranulins family. These molecules are abundant in the TME of most solid tumors, thus increasing the complexity of intracellular signaling networks involving RAGE [22–26].

Engagement of RAGE by its ligands initiates complex signaling pathways, including activation of NADPH oxidase, p21ras GTPase, several kinases (such as ERK1/2 (p44/p42) MAP kinases, p38 and SAPK/JNKMAP kinases, and phosphoinositol-3 kinase (PI3K)), and rho GTPases, as well as JAK/STAT pathways, with crucial downstream inflammatory consequences, including activation of NF-kB, AP-1, and Stat-3. Notably, activation of NF-kB up-regulates RAGE expression itself, thus generating a positive feed-forward loop, causing more inflammation [27].

Since the pioneering work of Taguchi et al. [28], who demonstrated that blockage of RAGE decreased tumor growth and metastases, the RAGE axis has emerged as a new actor in tumor biology, and its contribution to tumor growth and development has been widely documented in various types of cancer.

**Figure 2:**
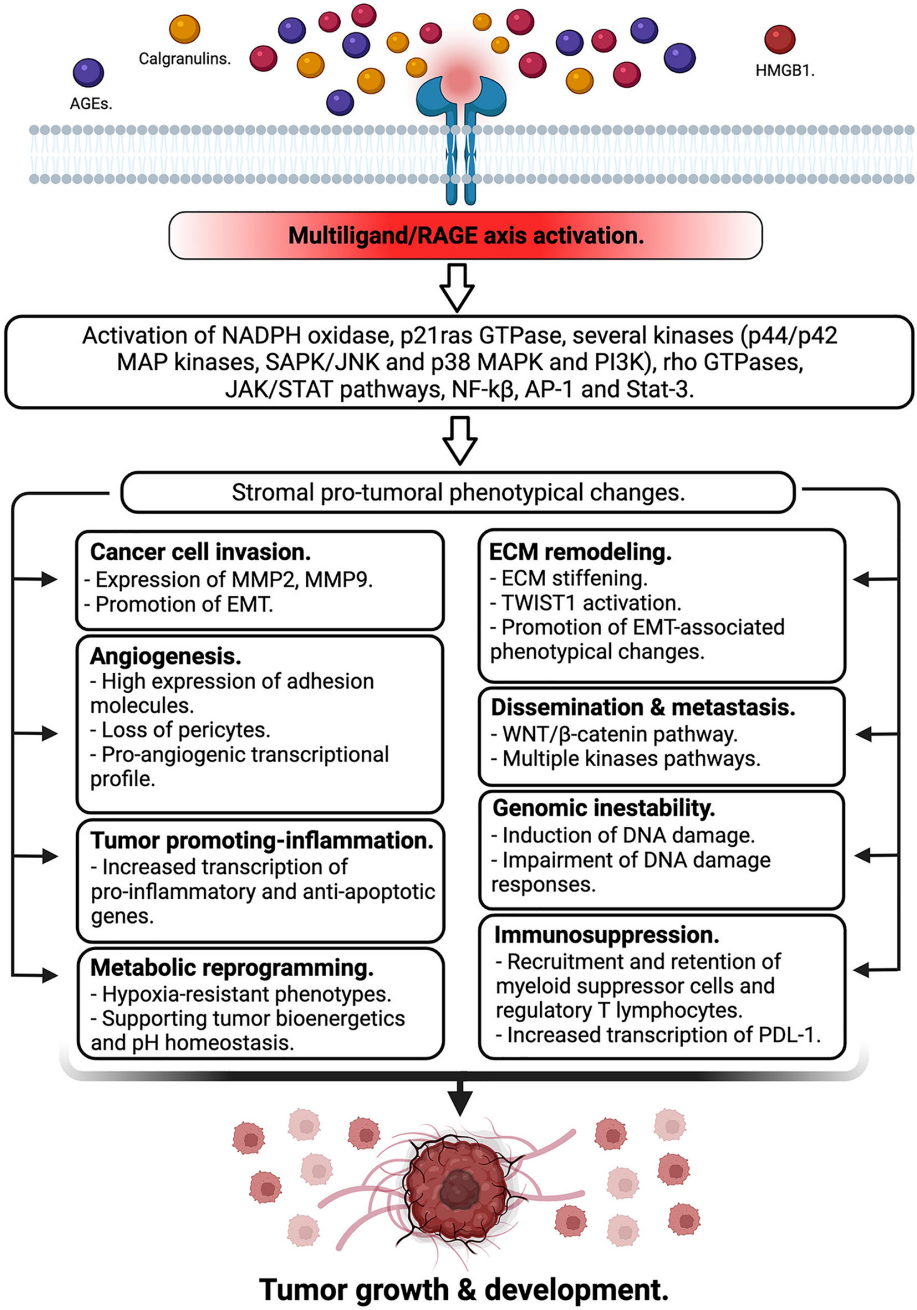
RAGE axis activation in the tumor microenvironment RAGE is either expressed by tumor cells or infiltrating immune cells in the tumor microenvironment. Many of its ligands are also abundantly present in the same tissue niche. The activation of this multilgand receptor triggers different signaling cascades promoting stromal pro-tumoral phenotypical changes mediated by a myriad of mechanisms supporting tumor growth and development.

In the present review, we highlight how the RAGE/multiligand axis has emerged as a relevant actor in tumor biology, based not only on the high diversity of signaling cascades triggered upon its activation, but also on the wide repertoire of ligands of this axis, as well as their relative abundance in the TME. We discuss how activation of the axis can promote numerous crucial steps during tumorigenesis, from genetic instability, fueling chronic inflammation and supporting many phenotypic changes in tumor cells favoring their growth and dissemination, to the onset of an immunosuppressive environment that restricts host immune responses, and thus support tumor growth and development (Figure 2).

## Cancer cell invasion

Epithelial–mesenchymal transition (EMT) is a form of trans-differentiation of epithelial cells into mesenchymal phenotypes, which plays a key role in many physiological processes, such as embryogenesis and tissue morphogenesis, wound healing, and regulation of stem cell behavior [29]; however, the onset of malignancy of many types of cancer cell, and quite possibly all of them, also depends on EMT activation [30]. This switch in cell phenotype and behavior is mediated by fine regulation of growth and transcription factors, as well as specific signaling pathways that respond to extracellular stimuli [31].

Activation of the RAGE/multiligand axis can strongly influence cell invasion through a wide variety of molecular signaling pathways, which converge in promoting cancer growth, invasion, and metastasis through enhancement of EMT [32–34]. Further, emerging studies have demonstrated effective repression of EMT in experimental RAGE knockout models, highlighting the central role of both AGEs and multiligand/RAGE axis activation in promoting enhanced EMT and contributing to highly invasive malignancies through multiple phenotype changes induced in the ECM, TME, and stromal cells [35,36]. Tumor-associated immune cells, as well as cancer cells themselves, can release high levels of the alarmin, HMGB1, within the TME of a wide variety of malignancies [37,38]. HMGB1 and its subsequent binding to RAGE can trigger the production of proinflammatory cytokines through NF-kB activation [39–42], as well as the expression of pro-invasive and proteolytic matrix metalloproteinases (MMPs), such as MMP2 and MMP9, leading to tumor invasion and metastasis [43].

The central role of the RAGE/HMGB1 axis in contributing to EMT in a PI3K/AKT-dependent manner widely documented in prostate cancer [44–46], and colorectal cancer [33].

In addition, overexpression of the RAGE ligands, S100A7, S100B, S100A4, and S100A8/A9, occurs in both triple-negative breast cancer and colon cancer. In each of these neoplasias, over-expression of these alarmins can increase RAGE expression and fuel an inflammatory milieu at the TME through NF-kB activation-dependent mechanisms, thus favoring invasive properties of these cancer cells [47,48].

## Dissemination and metastasis

The metastatic spread of malignant cells from a primary tumor to distant organs is considered the principal cause of cancer morbidity and lethality [49]. Cancer cell dissemination is supported by various cellular mechanisms, including neoplastic cell growth, local invasion of surrounding tissue, entry of circulating tumor cells within the microvasculature of the lymph and blood systems, escape from immune surveillance, and adaption to foreign microenvironments of distant niches, to facilitate cell proliferation and the formation of secondary macroscopic malignant tumors [50,51]. There are convincing data supporting a crucial role for RAGE and its ligands in facilitating tumor growth, progression, and metastatic spread of several types of malignant tumor [44–48,52].

RAGE engagement by HMGB1, S100A8/A9, or S100P proteins can activate different oncogenic pathways, supporting the metastatic spread of colorectal cancer cells. Hence, activation of RAGE by all of these ligands can trigger multiple signaling pathways, including K-RAS, ERK1/2 MAP kinases, p38 and SAPK/JNK MAP kinases, the WNT/β-catenin pathway, NF-κB, AP-1, and mTOR, causing downstream secretion of important inflammatory and apoptotic markers, as well as the upregulation of oncogenic miRNAs, such as miR-21 and miR-155 [53–57].

Interestingly, *in vitro* essays have revealed that activation of the RAGE/multiligand axis also significantly increases the metastatic potential of breast cancer cells, throughout activation of the RAGE/TLR4/MyD88/NF-kB signaling pathway, which results in elevated expression of MMP9, thus contributing to cancer migration and invasion by degrading ECM in patients with breast cancer [58].

The role of the RAGE axis on lung cancer remains controversial when compared with other cancers. Although RAGE is highly expressed in normal alveolar epithelium, it is surprisingly reduced in lung carcinomas. Some reports show that lung cancer progression may be enhanced by the RAGE downregulation in human lung carcinomas [59]. Novel experiments have demonstrated that RAGE activation promotes lung cancer metastasis *in vivo*, by supporting cell migration and EMT through ERK1/2-mediated activation of Snail, Slug, and Twist in lung adenocarcinoma cells [60]. This report has shed light on this controversy, based on the identification of a dual role of RAGE, which might cause growth inhibition in the early tumor formation while promoting EMT and providing a beneficial TME for tumorigenesis in lung adenocarcinoma.

## ECM remodeling in the TME

The ECM is a heterogeneous and well-orchestrated network of cell-secreted molecules that offer biochemical and structural support to cells, tissues, and organs [61]. In humans, the ECM is composed mainly of water, fibrous proteins, and various polysaccharides, providing both mechanical and biochemical support to tissues, and regulating diverse cellular functions, such as proliferation, survival, growth, differentiation, and maintenance of tissue homeostasis [62].

This complex network is considered highly dynamic, and undergoes continuous remodeling driven by close interplay among ECM components and surrounding cell and non-cellular elements [63]. During those different ECM interactions, several bidirectional signaling pathways are activated through mechanotransduction receptors, which can regulate cell behavior at local and systemic levels in response to modifications detected in the composition and stiffness of the ECM [64,65]. There is increasing evidence that ECM remodeling and stiffening act as key modulators of cell fate and function [66]. The role of tumor-associated ECM modifications in cancer cell biology has been extensively studied, particularly in most solid tumors where desmoplasia is a very common feature that leads to fibrosis through abnormal extracellular matrix (ECM) deposition, remodeling, and post-translational modifications. All these changes are crucial in the progression of the disease toward an increasingly aggressive and invasive tumor phenotype, mainly by forming a scaffold in the TME and contributing to all cancer hallmarks [67,68].

Strikingly, most ECM fibrous proteins are long-lived potential targets of AGE formation, resulting in ECM stiffening which generates mechanical cues that act on many stromal cellular components, including cancer cells, and thus stimulating cell transdifferentiation, EMT, and cell migration and invasion [69–71].

AGEs and AGEs precursors such as dicarbonyls are highly abundant in TME because of the ‘Warburg effect’ in cancer cells [19]. Glycation of ECM proteins mainly occurs at functionally significant arginine residues of arg-gly-asp (RGD) and gly-phe-hyp-gly-glu-arg (GFOGER) motifs generating marked structural distortion and loss of charge, and thus increasing the stiffness of the fibrous components of ECM, such as collagens [72].

The accumulation of highly reactive AGE precursors, such as MGO, and the increased rate of AGE formation not only mediates structural and mechanical changes in the ECM, but can also generate a reservoir of AGEs with the potential to trigger a multitude of pro-tumorigenic RAGE-dependent mechanisms, including altered cell adhesion, increased invasion, and enhanced migration, thus favoring cancer metastasis [73].

Recently, tumor ECM post-translational modifications derived from the accumulation of endogenous intermediates of AGEs, such as MGO, were reported to act as key players involved in cancer onset and progression [19], as well as migratory signaling pathway activation, thus favoring metastatic dissemination of breast and colorectal cancer cells [74,75].

In solid tumors, fibronectin and type I collagen are the most common and abundant fibrillar proteins found in cancer-associated ECM [76,77]. Their accumulation in tumor stroma creates a very dense network of ECM fibrillar proteins, which results from excessive fibrotic remodeling, largely mediated by myofibroblasts expressing alpha-smooth muscle actin. This remodeling leads to extensive modification of surrounding tumor cells and contributes to fibrotic changes in cancer cells, which enhances tumor cell aggression, promoting invasion and migration into stromal tissue [78,79]. Additionally, an extensive transformation of fibroblast-type cells to a myofibroblastic phenotype is also associated with increased tumor interstitial fluid pressure [80]. This biophysical alteration of the TME interferes with the transcapillary transport of therapeutic agents, decreasing tumor uptake of drugs, and consequently decreasing the efficacy of chemotherapy [81], particularly in cancer types with extensive desmoplastic reactions, such as breast and pancreatic cancer [82–84].

Furthermore, AGE-mediated cross-linking of load-bearing proteins leads to ECM stiffening, which promotes cancer cell survival as well as high rates of invasion, proliferation, and metastatic tumor cell interaction with the endothelium, and favors pro-angiogenic tumor phenotypes [73,85,86]. This AGE-induced ECM stiffness is associated with several phenotypic changes linked to the EMT, such as increased expression of vimentin and reduced expression of E-cadherin, which can contribute to the development of pre-metastatic niches in surrounding tissues, supporting the migration and invasion of tumor cells into future sites of metastasis, even before their arrival [79,86–88].

In addition, ECM stiffening can induce integrin-dependent phosphorylation events, resulting in the release of essential regulators of matrix-stiffness-induced EMT, such as the TWIST1 transcription factor, which is untethered from its cytoplasmic anchor, G3BP2, thus favoring its entrance to the nucleus to drive a transcriptional profile supporting cancer cell invasion in epithelial neoplasias [89]. Moreover, a novel cross-talk mechanism occurs, in which ECM stiffening mechano-sensitizes human malignant cells to drive invasiveness and migration in an EGFR-dependent manner. Squamous carcinoma cancer cells can respond to matrix stiffening, by increasing EGFR expression, thus sensitizing carcinoma cells to EGFR phosphorylation, resulting in increased actomyosin contractility and collective invasion [90].

## Inflammation in the TME

There is a growing body of evidence supporting a contribution of inflammation to the development of different malignancies through a complex network of mechanisms that represent an active landscape for tumor initiation, progression, and invasion [91–97]. Most cancer cells not only overexpress RAGE but also release high concentrations of its ligands [44,98–100]. These factors lead to increased survival of disseminated tumor cells and consequently increase the metastatic burden in a RAGE-dependent manner [101–103]. As mentioned above, activation of the AGE/RAGE axis triggers a robust pro-inflammatory response, and consequent increased leukocyte activation and apoptosis, enhanced desmoplastic reactions, and recruitment of stromal cells into the TME [104–109]. In this context, the contribution of NF-kB-dependent pathways to induction of pro-inflammatory genes is well-documented, and is key in fueling a pro-inflammatory milieu, in both tumor and tumor-associated cells, as well as surrounding host tissues [110–113]. Notably, a key consequence of RAGE-induced NF-kB activation is transactivation of genes encoding several pro-inflammatory factors, such as TNF-a, COX-s, iNOS, IL-1, and IL-6, as well as proangiogenic factors (vascular endothelial growth factor (VEGF)) and anti-apoptotic signals (BcL-X, BcL-2, XIAP) [114–119].

The increased rate of release of many RAGE ligands by cancer cells can act in both autocrine and paracrine dependent-manners on RAGE-positive cells at the tumor-host interface, to promote cancer cell survival [29,120,121].

A convincing body of evidence shows both increased expression of HMGB1 in several solid tumors and its critical role as an emerging prognostic factor in prostate cancer, breast cancer, and gastric cancer [122–125]. Biological responses downstream of HMGB1 are implicated in promoting tumor proliferation, migration, and invasion by stimulating production of pro-inflammatory cytokines through RAGE-dependent pathways [37]; however, HMGB1 can signal through TLRs (TLR2 and TLR4), as well as RAGE, thereby triggering NF-kB, STAT-3, and MyD88-dependent pathways and promoting inflammation and tumorigenesis [126–128]. Given the similarities between TLRs and RAGE and their signaling cascades, RAGE has been proposed as an emerging non-canonical Toll receptor [129]. Consequently, it is reasonable to consider that HMGB1-activation of both RAGE and TLRs can enhance the recruitment and assembly of homo- and hetero-oligomers, to strengthen pro-inflammatory responses in the TME and stimulate acquisition of a hypoxia-resistant phenotype in hepatocellular carcinoma and breast cancer cells [130,131]. The S100 protein family members, S100A8A/S100A9 [132], which form an S100A8/A9 heterodimeric complex able to interact with both TLR4 and RAGE on tumor cells, are also key in promoting RAGE-mediated inflammatory responses [133,134]. S100A8/A9/RAGE axis activation act as a novel pro-inflammatory signaling cascade in prostate, breast, and pancreatic cancers, triggering multiple downstream pro-inflammatory signaling pathways within the TME, thus promoting tumor cell survival, progression, and metastasis [135,136]. In addition, emerging clinical evidence suggests that tumor-associated S100 protein levels have potential as a new prognostic biomarker and therapeutic target in patients with breast and pancreatic cancers [136,137]. In addition, several studies support an association between NF-kB and hypoxia-inducible factor 1 (HIF-1), where NF-kB is a key transcriptional activator of constitutive HIF-1α, requiring basal NF-kB activity for HIF-1 protein accumulation under hypoxic conditions [138,139].

## Angiogenesis

The formation of new blood vessels from pre-existing vasculature is a major mechanism of vascularization during embryonic development and some physiological processes, such as wound healing; however, angiogenesis is also crucial in the delivery of oxygen and nutrients to cancer cells, which produce several proangiogenic factors to overstimulate angiogenesis and thereby support tumor survival, growth, and metastasis [140].

There is a compelling body of evidence supporting that activation of the multiligand/RAGE axis is important in tumor associated-angiogenesis modulation, by triggering upregulation of VEGF and MMP2, as well as disruption of VE-cadherin-catenin complexes, thereby favoring capillary tube formation [141,142]. Additionally, the excessive capillary tube formation induced through AGE-mediated RAGE signaling may also involve increased expression of several scavenger receptors, such as CD36, CD136, and LOX1, and their subsequent activation by AGEs [143]. The crucial role of RAGE signaling in tumor angiogenesis has been highlighted by studies using experimental methodologies aimed at both gene silencing and receptor blockade in different cancer types [144,145].

Strikingly, the pro-inflammatory signaling cascades induced by RAGE activation also directly reduce pericyte numbers, which in turn relieves the restriction of endothelial cell (EC) replication, and thus facilitates new blood vessel formation [146].

Tumor overexpression of the alarmin, HMGB1, and subsequent activation of RAGE signaling induces secretion of pro-inflammatory cytokines, such as TNF-α and IL-8, as well the expression of leukocyte adhesion molecules (ICAM1, VCAM1, and E-selectin) through NF-kB activation in ECs, thus stimulating EC proliferation and sprouting in vitro and neovascularization *in vivo* [147]. Notably, HMGB1 may also favor endothelial progenitor cell homing and increase their neovascularization capacity in a RAGE/TLR4-dependent manner, thus promoting tumor angiogenesis [148]. Recently, internalization of HMGB1 has been reported as a novel mechanism by which this alarmin induces angiogenesis in ECs through a RAGE-mediated pathway [149]. Hence, HMGB1/RAGE axis activation in ECs not only triggers both positive autocrine and paracrine mechanisms to promote a pro-angiogenic gene expression profile, [148–150] but also stimulates VEGF production by some pro-tumoral stromal cells, such M2 macrophages [151]. Furthermore, *in vivo* and *in vitro* data suggest that HMGB1 is crucial for the activation of a positive loop promoting the tumoral pro-angiogenic response by increasing the expression of both RAGE and TLR4, and consequently their pro-inflammatory signaling cascades, which perpetuate the transcription of pro-angiogenic and pro-inflammatory genes [150,152,153]. Activation of the multiligand/RAGE axis by members of the S100/calgranulin protein family contributes to fueling the pro-angiogenic tumoral phenotype [154]. S100A7-mediated RAGE activation is crucial in modulating pro-inflammatory and angiogenic pathways in many cancer types, particularly in cervical [101], breast [47,155,156], and esophageal squamous cell carcinoma [157]. During mammary tumorigenesis S100A7 increases the expression of reactive oxygen species (ROS) and VEGF by RAGE-dependent mechanisms, thus enhancing cancer cell progression by promoting oxidative stress responses and angiogenesis [47,158].

Metabolic disorders, such as obesity and insulin resistance, are associated with poor prognosis and development of highly aggressive breast cancer cells, and are thus critical factors mediating survival prognosis across all stages of breast cancer [159]. Furthermore, patients with these comorbidities show an altered expression profile of both RAGE and the IGF-1/IGF-1R axis, favoring STAT3-dependent transcriptional activation of the S100A7 gene, thus enhancing many mammary cell S100A7/RAGE-dependent pathways, such EC proliferation and angiogenesis [156].

The S100 protein family member, S100A4, is a key factor in several biological functions mediated by RAGE activation, which trigger pro-inflammatory and pro-angiogenic responses to stimulate tumor progression and invasion [27,154]. Interestingly, several growth factors, such as FGF2, may induce upregulation and release of S100A4 through FGFR1, thus favoring pro-angiogenic and pro-tumoral transduction pathways triggered through the S100A4/RAGE axis in triple-negative breast cancer cells [160].

The role of nitric oxide (NO) in tumor angiogenesis remains controversial. NO can mediate angiogenesis by direct and indirect mechanisms [161], while anti-angiogenic effects of NO have also been also reported [162]. This apparent incongruity may be attributable to differences in concentration or cellular compartment, as well as duration of exposure [163,164].

Due to the pro-inflammatory nature of the TME, many stromal cell types, including cancer cells, express inducible nitric oxide synthase (NOS-II), which is upregulated by activation of the RAGE axis [165,166]. Recent reports support a role for NOS-II-derived nitric oxide as an important mediator of tumor growth and vessel maturation [167,168]. Furthermore, NO can induce the synthesis and activation of HIF-1α, which in turn up-regulates VEGF [169].

Recent studies have demonstrated, using *in vitro* and *in vivo* assays, that variations in NO flux into the TME induced by NOS-II or TLR/RAGE agonists contribute to HIF-1α stabilization, thus leading to transcription of numerous pro-angiogenic target genes, including *VEGF* [170,171].

## Metabolic reprogramming

Cell proliferation is a crucial hallmark in cancer development and progression, and tumors are forced to reprogram metabolic pathways involved in nutrient uptake and metabolism, to fulfill their high-energy requirements, produce biomolecules on demand, and maintain the redox balance [172–175]. The RAGE axis has emerged as a crucial actor in reprogramming different metabolic pathways, which are essential to ensure cancer cell progress.

A distinctive feature of many cancer types is the presence of a hypoxic TME. During tumor development and progression, both tumor and stromal cells have restricted access to nutrients and oxygen, either permanently or transiently, mainly due to aberrant vascularization and poor blood supply. This hypoxic microenvironment can stimulate HIF-driven transcriptional responses, which are involved in numerous adaptive metabolic changes, such as the switch from oxygen-dependent mitochondrial oxidative phosphorylation to oxygen-independent glycolysis, thus increasing glucose consumption and pyruvate, lactate, and H^+^ production, to meet their energy requirements [175–177].

The RAGE axis has emerged as a relevant actor in the hypoxic microenvironment. In a hypoxic milieu, the abundance of RAGE ligands is increased, particularly HMGB1, which is released by either infiltrating leukocytes or cancer cells themselves, under hypoxic conditions [178].

Disturbances in intracellular calcium homeostasis are a hallmark of hypoxia, linked to HIF-1α expression and stabilization [179]. Hypoxia-mediated cell damage, as well as the activation of immune cells by inflammatory signals, allows the release of S100 proteins to the extracellular space as damage-associated molecular pattern (DAMP) molecules, also known as alarmins, and then they can be recognized by and activating RAGE-mediated signaling [22,23,180].

Interestingly, the RAGE ligand, S100A10, has been reported to accelerate aerobic glycolysis and tumor growth by activating mTOR signaling through a RAGE-dependent mechanism [181].

Cancer cells exhibit a particular glucose metabolism characterized by increased glucose uptake, accompanied by the overexpression of glucose transporters, which are hypoxia-responsive elements [182,183].

Furthermore, hypoxic tumor cells shows a metabolic shift from mitochondrial aerobic respiration to anaerobic glycolysis process, where, di-carbonyl compounds, a major precursor of AGE formation, are generated [184,185]. Consequently, hypoxia-driven AGE accumulation, favoring activation of RAGE-dependent signaling, has been extensively reported in cancer cells [186].

Furthermore, pyruvate kinase muscle isozyme M2 (PKM2) is a rate-limiting glycolytic enzyme that catalyzes the final step in glycolysis [187]. PKM2 interacts with HIF-1α [188] and activates transcription of glycolysis-related genes, such as glucose transporter 1, which increases glucose uptake, and lactate dehydrogenase A, which increases lactate production, leading to excessive lactate production and HMGB1 hyperacetylation and its subsequent release in the TME [189].

In addition to its contribution by increasing the bioavailability of RAGE ligands, hypoxia itself may increase RAGE expression, because the promoter region of the RAGE gene contains at least one functional hypoxia response element [190–192]. Consequently, different groups have reported increased RAGE expression in the hypoxic TME [193,194]. Furthermore, increased RAGE expression and signaling in a hypoxic environment can also result in RAGE-dependent activation of HIF-1α [193], thus generating a potent amplifying loop.

Monocarboxylate transporters (MCTs) are crucial cellular regulators of cancer pH homeostasis, particularly within tumor cells with high glycolysis rates [195,196], and their plasma expression and activities, particularly those of MCT1 and MCT4, require the presence of the chaperone, CD147 [197,198]. Notably, CD147 expression appears to be regulated by RAGE-mediated signaling, since blocking RAGE suppressed the induction of CD147 expression [199]. Considering the high activation levels of the RAGE axis in the TME, this may favor the bioavailability of this chaperone in this niche, thus supporting the efficient functioning of these crucial pH regulators in cancer cells.

Interestingly, Kang et al. proposed that inflammatory signals within the TME, such as HMGB1, are crucial for the promotion of tumor bioenergetics, and thus support tumor progression. In support of this, HMGB1 is reported to promote RAGE translocation to mitochondria, leading to enhanced complex I activity and increased ATP production, through an ERK1/2 phosphorylation-mediated mechanism [200].

Adipose tissue regulates physiological energy balance [201], acting as a complex organ that stores lipids in adipocytes as an energy source and can release them by responding to physiological demands [202].

Tumors, either locally or during metastatic dissemination, are related closely with adipose tissue. For many cancer types, and particularly for breast, prostate, and ovarian carcinomas, adipocytes from adipose tissue establish a cooperative cross-talk with cancer cells, where adipocytes provide adipokines and lipids to cancer cells, while stromal and immune cells from AT also secrete paracrine factors within the tumor microenvironment to promote cancer survival, proliferation, metastasis, and treatment resistance [202–206].

Emerging data support that an intense reprogramming of many metabolic activities occurs when cancer cells are exposed to hypoxic conditions [207]. One of these metabolic changes involves the increased utilization of lipids, and particularly the use of fatty acids (FAs) as a source of energy through β-oxidation [208–211]. Even with sufficient dietary lipid supply, cancer cells can synthesize most FAs *de novo* [212]; however, these capacities are sometimes insufficient, as in some aggressive cancers, where tumor cells take up extracellular FAs from surrounding adipocytes [213–217].

CD36, an FA translocase, is a crucial molecule in FA transport from adipocytes to cancer cells [218], and overexpression of CD36 is associated with tumor progression and metastasis [219,220]; crucially, CD36 is up-regulated by RAGE-mediated signaling [220,221].

## Immunosuppression in the TME

There is both experimental and clinical evidence that, while tumors grow, the stroma becomes in an immunosuppressive niche [221]. In this context, the contribution of the RAGE axis to immunosuppression in the TME has been extensively documented in recent years.

RAGE activation by members of the calgranulins family (S100A8 and S100A9) contributes to the recruitment and retention of myeloid suppressor cells, as well as increasing the expression of both the alarmins, S100A8/A9, and the activity of inducible nitric oxide synthase and arginase-1, and thus favors tumor growth and metastasis [222–224].

HMGB1 is present in high concentrations in the TME mainly due to its production or release by both tumor cells and infiltrating inflammatory cells, and thus favors the establishment of a highly immunosuppressive TME [37]. HMGB1 can promote the influx of myeloid suppressor cells into the TME as well as the recruitment and activation of regulatory T lymphocytes by RAGE-dependent mechanisms [225–227].

Tumor-associated macrophages, and particularly M2-type macrophages, play a significant immunosuppressive role by secreting immunosuppressive molecules, such as IL-10, TGF-β, and human leukocyte antigen G [228]. Interestingly, hypoxia and the concomitant presence of HMGB1, are reported to promote M2-macrophage retention in the tumor hypoxic core of the TME, through a RAGE-dependent mechanism, by decreasing both the expression of CCR2 and the migration capacity of M2 macrophages [107].

PD-L1 is an immune checkpoint protein that helps cancer cells to escape from immunosurveillance [229]. RAGE activation by HMGB1 promotes NF-κB- and IRF3-dependent transcription of PD-L1 [230].

## Cancer genomic instability

The genomic instability in cancer cells is crucial in generating intratumoral genetic heterogeneity, thus supporting the extensive phenotypic diversity observed in many cancer types [231]. The ROS produced by inflammatory cells in the TME represents a crucial source of DNA damage in both tumor and stromal cells. These TME changes caused by oxidative stress may contribute to tumor development and even tumor spreading [232]. Decades of both clinical and experimental research have demonstrated that ROS are essential mediators in tumor biology, by various mechanisms, including induction of mutations in tumor suppressor genes and oncogenes and the activation of various oncogenic signaling pathways, as well as oxidative inactivation of several DNA repair enzymes, among many others [233–235].

Since the pioneering work of Wautier et al., demonstrating that the engagement of RAGE by AGEs triggers ROS generation by NADPH oxidase activation [236], several reports have supported that NADPH oxidase activation is a crucial event in RAGE signaling. As AGE formation is increased under high ROS levels, and the subsequent activation of RAGE by AGEs also leads to ROS production, the onset of a vicious cycle is highly potentiated [27,237,238].

The capacity of ROS to induce DNA damage and affect the DNA damage responses, in particular, the formation of 8-oxo-7,8-dihydroguanine, the most prevalent purine base oxidation, with highly mutagenic potential [239], has been widely documented. Furthermore, increased ROS can also trigger genomic instability, leading to DNA double-strand breaks and altered repair capacity, due to the generation of dysfunctional DNA repair enzymes [240].

As the RAGE cytoplasmatic (ctRAGE) domain lacks kinase activity in all mammals cells, including malignant cells, the mammalian diaphanous-1 (mDia1) protein is used to integrate oxidative and signal transduction pathways when RAGE is engaged [241–243]. Hence, many ctRAGE dependent signaling pathways are regulated through mDia1 [244].

Very recently, a nuclear isoform of RAGE (approximately 64 kDa) was identified as a positive regulator of DNA double-strand break repair via the mechanism of homologous recombination [245]. As the membrane-bound isoform of RAGE is over-expressed in multiple human cancers, it is tempting to speculate that changes in the expression/trafficking of this isoform may occur during malignant transformation, leading to decreased DNA repair capacity and increased genomic instability.

## Concluding remarks

There are currently lines of evidence drawn from clinical and experimental data supporting the role of the RAGE/multiligand axis in remodeling the TME to support tumor growth and development, based on the positive impacts of activation of the axis on relevant processes in tumor growth and development. Most cancer types overexpress RAGE, and some of its ligands are highly abundant in the TME. Hence, the potential use of RAGE as a biomarker of prognosis, as well as the therapeutic use of blocking RAGE/multiligand signaling has emerged as an area of intense research, based on data raised from many approaches such as gene-silencing technologies, in vivo RAGE knockout models, and the use of aptamers, and small synthetic molecules.

Additionally, many efforts are further required to understand the clinical significance of different soluble variants of the receptor. A widely held view is that soluble RAGE fulfills a protective anti-inflammatory role by acting as a decoy receptor, binding RAGE ligands and thus blocking their interaction with the full-length receptor. Some promising results indicate that decreased sRAGE levels in patients may contribute to the progression of the disease. However, these findings require much research because many conflicting findings between different studies that have been reported could be related to the demographic, genetic, and health characteristics of the populations under investigation.

However, some questions remain without answering before the whole comprehension of the role of the RAGE/multiligand axis on tumor biology can be achieved. One crucial and intriguing issue is the cross-talk of tumor cells with infiltrating immune cells and other stromal cells, and the characterization of all regulation mechanisms of the RAGE axis requires further investigation. Finally, intense research efforts are required to achieve an integrative comprehension of the consequences of proteome glycation in both cancer and stromal cells.

## Abbreviations

EC: endothelial cell
FA: fatty acid
HMGB1: high mobility group box 1
MCT: monocarboxylate transporter
NO: nitric oxide
PKM2: pyruvate kinase muscle isozyme M2
RAGE: receptor for advanced glycation end-products
ROS: reactive oxygen species
